# Repair by a molecular DNA ambulance

**DOI:** 10.18632/oncotarget.5140

**Published:** 2015-08-10

**Authors:** Daniel K.C. Chung, Karim Mekhail

**Affiliations:** Department of Laboratory Medicine and Pathobiology, Faculty of Medicine, University of Toronto, Toronto, Canada

**Keywords:** Chromosome Section, double strand break, DNA damage, kinesin, nuclear pore complex, nuclear envelope

Most of the genome of a eukaryotic cell is located in its nucleus, which is a ball-like entity defined by a membrane bilayer known as the nuclear envelope. Constitutive physical interactions between certain chromosomal domains and inner nuclear membrane proteins can directly promote genome stability and cellular lifespan by preventing aberrant DNA recombination events [1, 2]. In addition, DNA loci experiencing DNA double-strand breaks (DSBs) exhibit increased interactions with nuclear pore complexes at the nuclear envelope and this is thought to contribute to DNA repair [3, 4, 5]. That a change in the subnuclear positioning of a damaged DNA locus may contribute to its repair has been observed in various organisms including yeast and human [3-6]. DSBs changing location may be escaping subnuclear neighbourhoods that are not conducive to repair, moving to specialized regions that directly promote repair, and/or searching for homologous DNA sequences to serve as donors for repair. How DSBs move within the eukaryotic nucleus and what DNA repair pathways are engaged via this movement is unclear.

We recently utilized the power of yeast genetics in order to address these questions. We assessed the ability of cells to survive DSBs precisely induced at different locations across the genome and analyzed the chromosomes of cells surviving the DNA break [7]. This analysis revealed that DSBs close to linear chromosome ends, or telomeres, are preferentially repaired via an error-prone type of homologous recombination called break-induced replication (BIR) [7]. Essential to this repair process were inner nuclear membrane proteins that typically work to tether telomeres to the nuclear envelope. Also critical to DSB survival was a particular nuclear pore complex. Abrogating the inner nuclear membrane proteins but not the nuclear pore complex released telomeres from the nuclear envelope in the absence of DNA damage. This is consistent with the fact that yeast telomeres are typically arranged in a handful of clusters abutting the inner nuclear membrane but away from nuclear pore regions. Interestingly however, genetic and molecular biology experiments revealed that DSB induction greatly increases physical interactions between the damaged chromosome ends and nuclear pore complexes. This increased interaction is dependent on perinuclear telomere tethers as well as a kinesin motor protein complex called Kinesin-14. This BIR-dependent DNA repair process was promoted via disruption of chromatin silencing but repressed upon abrogation of chromatin remodelling, DNA damage responses, or microtubule stability. Interestingly, artificially targeting DNA loci to nuclear pore complexes via the use of so-called ‘DNA zip codes’ hyper-activated this DNA repair process.

Importantly, repair of a DSB induced at a locus located more internally on the same chromosome arm did not require perinuclear telomere tethers, motor proteins, nuclear pore complexes, or the homologous recombination machinery [7]. Instead, repair of this control site was dependent on non-homologous end joining. In contrast, strong DNA resection near chromosome ends ensures the engagement of homologous recombination/BIR. We also found that kinesin-14 and nuclear pore complexes, but not perinuclear telomere tethers, cooperate to repair nontelomeric DSBs that are repairable via BIR. Monitoring DSB mobility profiles in living cells in combination with molecular biology experiments indicates that Kinesin-14 allows for the transient relocation of DSBs to nuclear pore complexes for repair.

BIR is physiologically important for the maintenance of telomeres in the absence of telomerase via a recombination-based mechanism that is akin to the ‘alternative lengthening of telomeres’ seen in telomerasenegative human cancers. Interestingly, we found that Kinesin-14 promotes recombination-based telomere maintenance and limits senescence in the absence of telomerase [7]. Thus, motor proteins can help cells survive stressful events such as DSBs or telomerase loss by engaging DNA recombination pathways that actually promote the propagation of a compromised genome in a cell population, a scenario that commonly leads to cancer.

Taken together, our findings indicate that motor proteins can act like a ‘DNA ambulance’ that helps transport damaged DNA to ‘DNA hospitals’, or NPCs [7]. Importantly, this hospital helps repair damaged DNA via an error-prone process that promotes cell survival at the expense of genome fidelity. On a broader level, it is well established that motor proteins perform various critical roles in the cell. This includes the transport of vesicles in the cytoplasm and the movement of chromosomes during cell division. Our study expands the functional repertoire of motor proteins to nuclear DNA repair and the movement of interphase chromosomes. Our work also raises many new and important questions. For example, how do microtubules cooperate with motor proteins to mediate DNA repair? Can motor proteins move interphase chromosomes to regulate other DNA related processes such as replication and gene expression? Can motor proteins transport other nuclear nucleic acids including various types of RNA molecules? If some motor proteins promote cancer by ensuring the propagation of a compromised genome, can this help us develop novel anti-cancer drugs? In conclusion, since most of the macromolecules linked to this novel DNA repair mechanism are evolutionarily conserved, we expect that similar DNA movement and repair processes exist in various organisms.
